# Supplementary material for: Regulation of RXR-RAR Heterodimers by RXR- and RAR-Specific Ligands and Their Combinations
Source: Cells. 2019 Nov 5;8(11):1392. doi: 10.3390/cells8111392 (PMC6912802; doi:10.3390/cells8111392)
Supplement: Supplementary file 1 [file cells-08-01392-s001.pdf]

Supplementary Materials

Regulation of RXR-RAR heterodimers by RXR- and RAR- specific ligands and their combinations

Albane le Maire, Catherine Teyssier, Patrick Balaguer, William Bourguet and Pierre Germain

Supplementary Table 1. Data collection and refinement statistics.

**Complexe**	**RXRα/LG754/TIF2**	**RARβ/LG754/SRC1**
**PDB ID**	**6STI**	**6SSQ**
**Resolution range**	46.6 - 1.9 (1.96 - 1.9)	67.2 - 2.3 (2.38 - 2.3)
**Space group**	P 43 21 2	P 21 21 21
**Unit cell**	65.96 65.96 111.13 90 90 90	58.21 85.30 109.29 90 90 90
**Total reflections**	200743 (18915)	286909 (28587)
**Unique reflections**	20270 (1975)	24860 (2449)
**Multiplicity**	9.9 (9.6)	11.5 (11.7)
**Completeness (%)**	99.95 (99.95)	99.94 (99.84)
**Mean I/sigma(I)**	51.1 (7.9)	46.5 (3.3)
**Wilson B-factor**	18.30	31.21
**R-merge**	0.571 (0.814)	0.759 (2.08)
**R-meas**	0.602 (0.860)	0.795 (2.18)
**R-pim**	0.188 (0.272)	0.231 (0.631)
**CC1/2**	0.72 (0.74)	0.71 (0.44)
**CC***	0.92 (0.92)	0.91 (0.78)
**Reflections used in refinement**	20263 (1974)	24844 (2446)
**Reflections used for R-free**	1012 (111)	1234 (131)
**R-work**	0.156 (0.181)	0.164 (0.216)
**R-free**	0.172 (0.225)	0.209 (0.268)
**CC(work)**	0.83 (0.86)	0.89 (0.65)
**CC(free)**	0.79 (0.86)	0.90 (0.63)
**Number of non-hydrogen atoms**	2103	4215
**macromolecules**	1783	4012
**ligands**	33	77
**solvent**	287	126
**Protein residues**	225	509
**RMS(bonds)**	0.011	0.008
**RMS(angles)**	1.27	1.21
**Ramachandran favored (%)**	97.3	97.2
**Ramachandran allowed (%)**	2.7	2.6
**Ramachandran outliers (%)**	0.00	0.20
**Rotamer outliers (%)**	0.5	0.00
**Clashscore**	6.55	4.86
**Average B-factor**	22.0	44.0
**macromolecules**	20.4	44.0
**ligands**	16.3	50.3
**solvent**	32.1	40.8

Statistics for the highest-resolution shell are shown in parentheses.

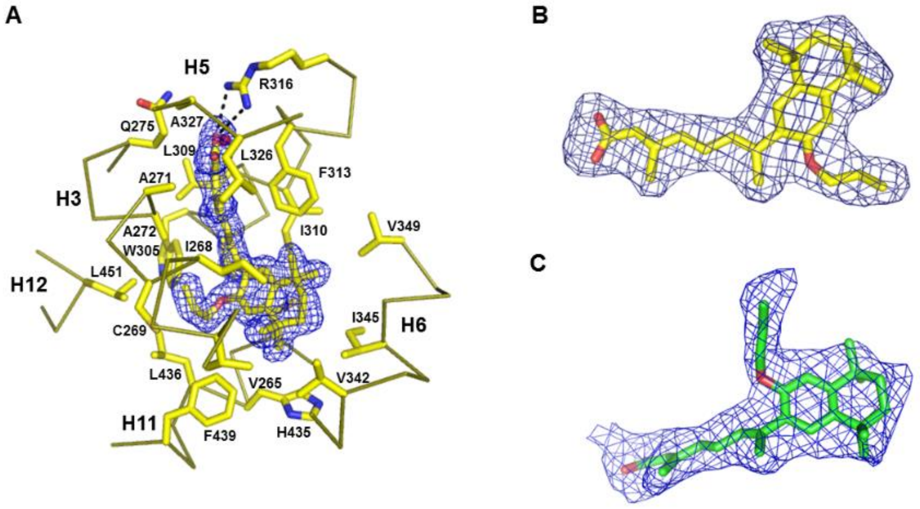

Supplementary Figure 1: A. LG754 is shown in its 2Fo-Fc electron density map contoured at 2 σ. Side chains of RXRα LBP residues in interaction with LG754 are shown as yellow sticks. **B. and C.** LG754 modelled into the difference density of each RAR molecules of the asymmetric unit of the RARβ crystal structures. The omit Polder map contoured at 3 σ are shown.

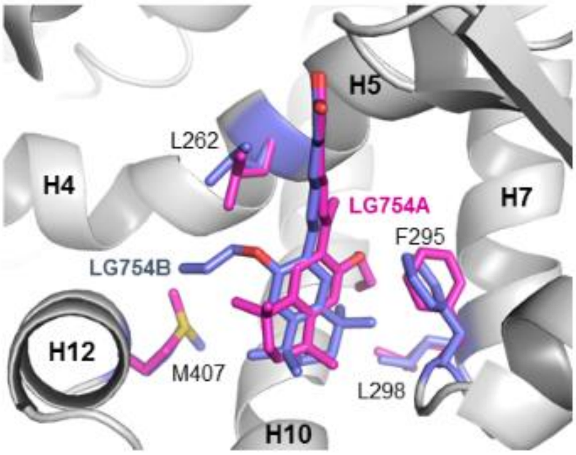

Supplementary Figure 2: Close-up view of the superposition of LG754 in the RARβ LBP from the two complexes present in the asymmetric unit. Side chains of the amino acids that have different positions in both complexes are shown in sticks representation. The main differences observed concern M407 and F295 that are closed to the propoxy group of LG754.
